# Vitrectomy combined with glial tissue removal at the optic pit in a patient with optic disc pit maculopathy: a case report

**DOI:** 10.1186/1752-1947-2-103

**Published:** 2008-04-07

**Authors:** Makoto Inoue, Kei Shinoda, Susumu Ishida

**Affiliations:** 1 Department of Ophthalmology, Keio University School of Medicine, Shinanomachi, Shinjuku-ku, Tokyo 160-8582, Japan; 2 Kyorin Eye Center, Kyorin University School of Medicine, Shinkawa, Mitaka, Tokyo 181-8611, Japan

## Abstract

**Introduction:**

We present a case of a man with optic disc pit maculopathy, whose vision improved after vitrectomy combined with glial tissue removal from the optic pit area, and without the use of photocoagulation.

**Case presentation:**

A 45-year-old man complained of blurred vision, and ophthalmoscopy revealed a retinal detachment and retinoschisis extending from an optic disc pit through the macula in his left eye. He was diagnosed with optic disc pit maculopathy, and vitrectomy was performed. A posterior vitreous detachment was created, glial tissue at the optic pit was removed, and octafluoropropane (C_3_F_8_) was injected as a gas tamponade. The retinal detachment and retinoschisis disappeared after six months, and vision improved to 20/20 without any visual field defects (Goldmann perimetry). A cataractous lens was extracted 2 years after the vitrectomy, and vision has remained 20/20 for 10 years without any recurrence.

**Conclusion:**

The removal of glial tissue during vitrectomy may be beneficial in patients with optic disc pit maculopathy.

## Introduction

Optic disc pit maculopathy is characterized by a congenital optic disc pit associated with a macular detachment and retinoschisis [1]. Focal laser photocoagulation of the temporal juxtapapillary retina or vitrectomy combined with a gas tamponade has been reported to be effective in treating this syndrome [1,2]. However, a relatively high incidence of recurrence has been reported after laser treatment alone [1]. A combination of both procedures has recently been reported to be an effective treatment [3].

Glial tissue is occasionally seen in the optic disc pit of patients with an optic disc pit or coloboma, but the reason for its development is unknown. We report a case of a man with optic disc pit maculopathy that was successfully treated using vitrectomy combined with the removal of glial tissue from the optic disc pit followed by the use a gas tamponade.

## Case presentation

**Figure 1:**
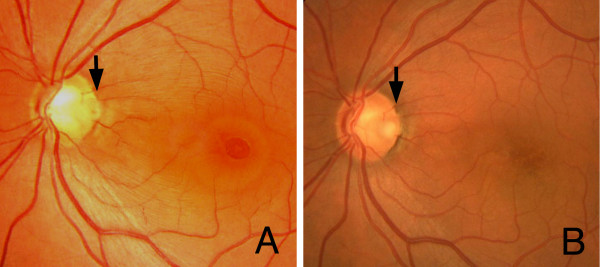
**Fundus photograph of the left eye before and after vitreous surgery**. (A) Retinal detachment and retinal schisis can be seen with a double ring apparatus. Glial tissue can be seen at the optic pit (arrow). (B) Fundus photograph of the left eye four years after vitreous surgery. Retinal detachment and retinoschisis are absent and the excavation of the optic disc is clearly seen after removal of the glial tissue (arrow).

**Figure 2:**
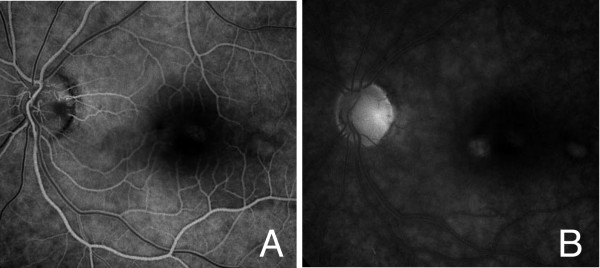
**Fluorescein angiograms of the left eye**. (A) Fluorescein angiogram in the early phase showing hypofluorescence at the optic pit and many hyperfluorescence spots can be seen in the macular lesion.(B) The hypofluorescence at the optic pit and glial tissue turned to hyperfluorescence with weak dye leakage in the late phase.

A 45-year-old Japanese man visited our clinic in September 1996 complaining of decreased vision in his left eye. His vision was decreased to 20/200, and ophthalmoscopy showed a retinal detachment involving the macula and a retinoschisis that extended from the optic disc pit through the macula in the left eye (Figure 1A). Glial tissue was seen at the optic disc pit but a retinal tear was not seen. Fluorescein angiography showed hypofluorescence of the optic disc pit, and multiple hyperfluorescent spots in the area of the macular lesion (Figure 2), but with no dye leakage. In the late phase, the optic disc pit and glial tissue became hyperfluorescent with mild dye leakage. He was diagnosed with optic disc pit maculopathy and vitrectomy was recommended.

After obtaining informed consent, vitrectomy was performed. A posterior vitreous detachment (PVD) was created by suction with a vitreous cutter until the 'fish-strike sign' was no longer seen. However, the vitreous cortex remained firmly attached at the optic disc pit. Neither condensed vitreous strands nor a residual Cloquet's canal was observed. It was decided intra-operatively that the glial tissue at the edge of the optic pit should be removed in order to remove the vitreous traction completely. Tapered forceps with a fine tip were used to avoid contact with the neural tissue at the edges of the optic pit. During this procedure, it was noted that the glial tissue was firmly attached to the temporal wall of the optic pit. An excavated space at the bottom of the optic pit was then clearly observed after removal of the tissue.

The vitrectomy was completed with a 14% octafluoropropane (C_3_F_8_) gas tamponade, and the patient was instructed to maintain a face-down position for a week. Under these conditions, the retinal detachment and retinoschisis gradually decreased, and the retinal detachment and retinoschisis were absent six months postoperatively (Figure 1B). Vision improved to 20/20 without any visual field defects (Goldmann perimetry).

**Figure 3:**
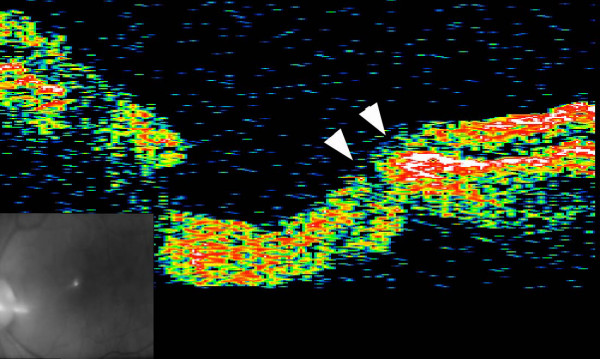
**OCT image seven years after vitrectomy**. Cross section of the temporal side of the optic disc showing two channels (arrowheads) connecting the vitreous cavity to the longitudinal space of the optic nerve and to the intraretinal space. The exit to the vitreous cavity is closed.

The patient's vision deteriorated to 20/40 owing to a nuclear sclerosis cataract two years after the vitrectomy, and the lens was extracted. Vision has remained 20/20 for 10 years without any recurrence of the retinal detachment or retinoschisis. Optical coherence tomography (OCT) at this time did not detect a retinal detachment or retinoschisis, but two channels were seen running from the vitreous cavity to the longitudinal space of the optic nerve, possibly the subarachnoid space and the intraretinal space. The exit of these channels to the vitreous cavity was closed (Figure 3).

## Discussion

The mechanism causing optic disc pit maculopathy has been considered to be vitreous traction on the optic disc pit. Bonnet [4] reported that 25 eyes with a macular detachment associated with an optic disc pit did not have a PVD, and two of the eyes had a reattachment of the macula after the development of a spontaneous PVD. Akiba et al [5] noted a residual Cloquet's canal moving in concert with a pulsating translucent membrane over the optic disc pit to cause anterior-posterior vitreous traction. However, the source of the subretinal fluid that causes the macular detachment and retinoschisis is still controversial, that is, whether it is vitreous fluid or cerebrospinal fluid [6].

The effectiveness of vitreous surgery to create a PVD around the optic disc with or without laser treatment has been examined [2,3]. The serous macular detachment associated with optic pits probably has a rhegmatogenous component. Thus, Postel et al [6] described a hole or tear in the diaphanous tissue overlying the optic pit in all of their seven cases. Todokoro et al [7], using careful OCT examinations, observed a cystic cavity covered with a superficial layer of optic disc tissue in a patient with a retinal detachment and retinoschisis that might correspond with the translucent membrane over the optic disc pit.

OCT was not performed preoperatively in our case, and the connection between the glial tissue and the optic disc pit was only detected intra-operatively. We suggest that the glial tissue might have developed after continuous vitreous traction of the vitreous strand attached to the optic disc pit. Thus, removal of the glial tissue might have made it possible to remove the vitreous traction completely, or to seal the retinal break at the optic disc pit by removing the translucent membrane and glial tissue associated with the wound healing process to close the two intraretinal channels seen in the OCT images. On the other hand, the procedures might have enlarged the retinal break in the pit or damaged the nerve fibres at the optic disc. A permanent escape may be produced mechanically by displacement of the fluid during vitreous surgery and the use of a gas tamponade, and the retina flattens with time even though the flow from the subarachnoid space remains constant. A reservoir for the fluid from the subarachnoid space might be the vitreous cavity with an egress into it provided by some retinal fenestration near the optic nerve head [8].

We cannot make a strong conclusion on the efficacy of the removal of the glial tissue based on a single case. However, considering the absence of any side-effects, such as visual field defects or decreased vision, removal of glial tissue may be beneficial in selected cases. Additional studies with larger samples are needed to evaluate the efficacy of glial removal.

## Conclusion

The removal of glial tissue during vitrectomy may be beneficial in patients with optic disc pit maculopathy.

## Competing interests

The author(s) declare that they have no competing interests.

## Authors' contributions

MI evaluated the patients and performed vitreous surgery. KS and SI reviewed the manuscript.

## Consent

Written informed consent was obtained from the patient for publication of this case report and accompanying images. A copy of the written consent is available for review by the Editor-in-Chief of this journal.

## References

[B1] Gass JD Serous detachment of the macula. Secondary to congenital pit of the optic nerve head Am J Ophthalmol 1969 67 821 841 5795739 10.1016/0002-9394(69)90075-0

[B2] Hirakata A Okada AA Hida T Long-term results of vitrectomy without laser treatment for macular detachment associated with an optic disc pit Ophthalmology 2005 112 1430 1435 10.1016/j.ophtha.2005.02.013 16024082

[B3] Garcia-Arumi J Guraya BC Espax AB Castillo VM Ramsay LS Motta RM Optical coherence tomography in optic pit maculopathy managed with vitrectomy-laser-gas Graefes Arch Clin Exp Ophthalmol 2004 242 819 826 10.1007/s00417-004-0897-8 15069565

[B4] Bonnet M Serous macular detachment associated with optic nerve pits Graefes Arch Clin Exp Ophthalmol 1991 229 526 532 10.1007/BF00203315 1765292

[B5] Akiba J Kakehashi A Hikichi T Trempe CL Vitreous findings in cases of optic nerve pits and serous macular detachment Am J Ophthalmol 1993 116 38 41 8328541 10.1016/s0002-9394(14)71741-6

[B6] Postel EA Pulido JS McNamara JA Johnson MW The etiology and treatment of macular detachment associated with optic nerve pits and related anomalies Trans Am Ophthalmol Soc 1998 96 73 88 10360283 PMC1298389

[B7] Todokoro D Kishi S Reattachment of retina and retinoschisis in pit-macular syndrome by surgically-induced vitreous detachment and gas tamponade Ophthalmic Surg Lasers 2000 31 233 235 10847502

[B8] Hotta K Unsuccessful vitrectomy without gas tamponade for macular retinal detachment and retinoschisis without optic disc pit Ophthalmic Surg Lasers Imaging 2004 35 328 331 15305558

